# Fms-like tyrosine kinase-3 ligand increases resistance to burn wound infection through effects on plasmacytoid dendritic cells

**DOI:** 10.1186/s12865-016-0188-2

**Published:** 2017-02-22

**Authors:** Leon Bae, Julia K. Bohannon, Weihua Cui, Monika Vinish, Tracy Toliver-Kinsky

**Affiliations:** 1 Department of Biochemistry and Molecular Biology, University of Texas Medical Branch, Galveston, USA; 2 Department of Anesthesiology, Vanderbilt University Medical Center, Nashville, USA; 3 Department of Anesthesiology, University of Texas Medical Branch and Shriners Hospitals for Children, 301 University Boulevard, Galveston, TX 77555-0877 USA

**Keywords:** Thermal injury, Wound infection, Dendritic cell, Neutrophil, Innate immunity

## Abstract

**Background:**

Patients experiencing large thermal injuries are susceptible to opportunistic infections that can delay recovery and lead to sepsis. Dendritic cells (DC) are important for the detection of pathogens and activation of the innate and acquired immune responses. DCs are significantly decreased in burn patients early after injury, and the development of sepsis is associated with persistent DC depletion. In a murine model of burn wound infection, the enhancement of DCs after injury by treatment with the DC growth factor Fms-like tyrosine kinase-3 ligand (FL) enhances neutrophil migration to infection, improves bacterial clearance, and increases survival in a DC-dependent manner. FL expands the production of both conventional DCs (cDC) and plasmacytoid DCs (pDC). It has been established that cDCs are required for some of the protective effects of FL after burn injury. This study was designed to determine the contribution of the pDC subset.

**Methods:**

Mice were subjected to full-thickness scald burns under deep anesthesia and were provided analgesia. pDCs were depleted by injection of anti-plasmacytoid dendritic cell antigen-1 antibodies. Survival, bacterial clearance, and neutrophil responses in vivo and in vitro were measured.

**Results:**

Depletion of preexisting pDCs, but not FL-expanded pDCs, abrogated the beneficial effects of FL on survival, bacterial clearance, and neutrophil migration in response to burn wound infection. This requisite role of pDCs for FL-mediated enhancement of neutrophil migratory capacity is not due to direct effects of pDCs on neutrophils. cDCs, but not pDCs, directly increased neutrophil migratory capacity after co-culture.

**Conclusions:**

The protective effects of FL treatment after burn injury are mediated by both pDCs and cDCs. Pharmacological enhancement of both DC subtypes by FL is a potential therapeutic intervention to enhance immune responses to infection and improve outcome after burn injury.

## Background

Patients experiencing large thermal injuries are susceptible to opportunistic infections, sepsis, and associated complications. Despite the widespread use of antibiotics, infection remains the leading cause of death among severely burned patients, necessitating the need of immune-modulating therapies [1]. Wounds and invasive instrumentation placed during acute care increase the risk of exposure to microorganisms. Furthermore, cells of both the innate and adaptive immune systems often experience debilitated or dysregulated functions following burn injury. For example, neutrophil chemotaxis is impaired in burn patients [2, 3]. Natural Killer (NK) cells have diminished killing capabilities, and macrophages can become inflammatory and lose phagocytic properties and the ability to eliminate foreign pathogens [4, 5]. Additionally, there is a decrease in the production of cytokines such as IFN-γ, IL-12, and IL-2 that are important mediators of Th1 responses in acquired immunity [6]. Dendritic cells (DCs) are important for the initiation of both the innate and acquired immune responses, through antigen presentation and secretion of IL-2, IL-12, IFN-γ and other cytokines that activate leukocytes to respond to infection. Burn patients have a significant decrease in circulating DCs early after injury and development of sepsis is associated with persistent DC depletion over the first 2 weeks after injury [7]. Thus, restoration of DC numbers and functions after burn injury may restore appropriate immune responses and prevent burn-associated infections.

Fms-like tyrosine kinase-3 ligand (FL) has been used experimentally to increase DC numbers and functions in models of severe burn injury [8]. The FL receptor, Fms-like tyrosine kinase-3 receptor (Flt3R), is expressed by multipotent progenitor cells and common lymphoid progenitor cells in the bone marrow. It is part of the tyrosine kinase class III family, and is essential for DC development. Binding of FL to Flt3R triggers progenitor cell proliferation and subsequent differentiation. Differentiation into non-DC cell types is associated with loss of Flt3R expression; however, DCs maintain expression of Flt3R after differentiation. In vivo administration of exogenous FL induces a significant expansion of both conventional DCs (cDCs) and plasmacytoid DCs (pDCs) in blood and lymphoid tissues of humans and mice [9–12]. Following thermal injury in mice, FL treatment increases DC numbers, enhances DC function, and increases survival in response to *Pseudomonas aeruginosa* infection in a DC-dependent manner. Enhanced bacterial clearance and survival are also dependent upon neutrophils, whose migration to sites of infection is increased, in a DC-dependent manner, by FL treatment [13]. The effects of FL on neutrophils are indirect and mediated by FL-enhanced DCs. Neutrophils do not express the Flt3R and FL enhancement of neutrophil migratory capacity is absent in DC-depleted mice. Furthermore, the direct exposure of neutrophils to FL-treated DCs in co-cultures enhances the chemotactic capacity of neutrophils [13]. Thus, the enhancement of DCs by FL treatment after burn injury is responsible for the improved neutrophil-mediated clearance of subsequent infections.

DCs consist of two principal and distinct subtypes. cDCs (MHC II^+^/CD11c^high^/B220^−^) are recognized as major initiators of effector cell activation in response to infection, through antigen recognition, cytokine production, and antigen presentation. pDCs (mPDCA-1^+^/B220^+^/ CD11c^low^ in mice), mostly known for IFN-α production in response to viral infection, can also induce immune responses [14]. pDCs are mobilized by inflammation and can enter lymph nodes directly from the circulation through high endothelial venules [15]. Although steady state pDCs are not as efficient as cDCs at the activation of T cells, activated pDCs can present antigen and induce T cell polarization [16]. There is increasing evidence that pDCs also play an important role in promoting cDC functions that activate immune responses. For example, pDCs are required by cDCs for efficient activation of T cells. By CD2-CD2L interactions, pDCs contact cDCs. Through subsequent CD40L-CD40 interactions, pDCs can help cDCs to generate anti-viral cytotoxic T cells and to produce IL-12 in response to CpG stimulation [17, 18].

Prior studies examining the effects of FL and DCs on responses to infection after burn injury focused on total (CD11c^+^) DCs and did not distinguish between the DC subtypes. This study was designed to investigate the contribution of pDCs to the protective effects of FL treatments after burn injury. Here we report that the presence of pDCs during in vivo treatment is required for FL to increase resistance to infection after burn injury. Depletion of pre-existing pDCs (prior to FL-induced expansion) in a murine burn wound infection model leads to significant loss of protective functions otherwise conferred by FL treatment. Furthermore, the FL-induced in vivo enhancement of neutrophil migration and bacterial clearance is dependent on pDCs, but through indirect effects on neutrophils.

## Results

### FL-mediated resistance to burn wound infection is dependent upon the presence of pDCs during treatment

**Fig. 1:**
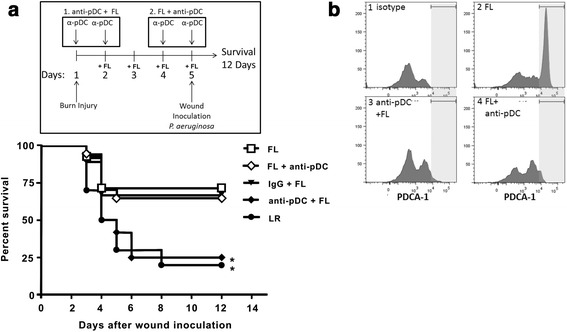
pDCs are essential for FL-mediated protection against a burn wound infection. **a** Timeline shows experimental protocols: Mice received burn injury on day 1 and FL treatment on days 2–5. Mice either received pDC depletion antibodies on days 1 and 2 (anti-pDC + FL) or on days 4 and 5 (FL + anti-pDC). IgG control mice received nonspecific IgG on days 1 and 2. Wounds were inoculated on day 5. Survival was monitored for 12 days following inoculation. *significantly different from FL, FL + anti-pDC, *p* ≤0.05, *n* = 9–17 mice per group. **b** Spleen and lymph nodes cells were stained and analyzed by FACS to confirm depletion of pDCs by anti-pDCA1 antibodies. Histograms are representative of staining in 3 mice/group and show PDCA-1 staining (light grey highlighted area) in CD45 (B220) + cells

As previously reported [8], treatment of mice with FL after burn injury significantly improved survival following a lethal *P. aeruginosa* burn wound infection. Survival in control (LR)-treated mice was 20% at 12 days post-inoculation, whereas survival in FL-treated mice was 71% (Fig. 1a). However, elimination of pDCs by injection of anti-PDCA-1 antibodies *prior to* FL treatment reduced survival to 25%. Injection of control IgG prior to initiation of FL treatment did not affect survival, which was 67% in this antibody control group. However, survival in mice depleted of pDCs *after* FL treatment, during wound infection, was 65% and similar to that in FL-treated control mice. Flow cytometric analysis of pDC markers (pDCA-1, B220) confirmed effective depletion of pDCs under both protocols (pDC depletion prior to and after FL treatment, Fig. 1b). This indicates that *pre-existing* pDCs, and not FL-expanded DCs, are necessary for FL to increase survival upon subsequent infection.

**Fig. 2:**
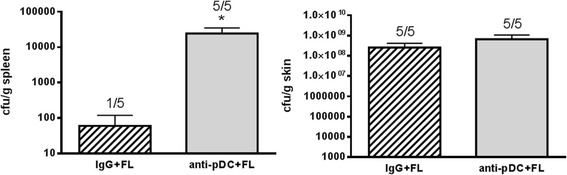
pDCs are required for FL to prevent bacterial dissemination from the wound. Spleens and wounds were harvested 3 days following wound inoculation, homogenized and cultured for determination of bacterial counts. Numbers over the graphs represent the number of mice with positive tissue cultures of the total number examined. *significantly different from IgG + FL, *p* ≤0.05, *n* = 5 mice per group

Increased survival in FL-treated mice is associated with a decrease in systemic dissemination of bacteria from infected burn wounds [8]. Since mortality was increased in FL-treated mice that had been depleted of pre-existing pDCs, bacterial dissemination after wound infection was examined in this group and compared to that in the control IgG- and FL-treated group. As expected, bacterial counts in the spleens of pDC-depleted FL-treated mice were significantly higher than in the control FL-treated mice (Fig. 2). Bacterial counts in the control FL group were detected in only one mouse and were negligible, but 100% of the pDC-depleted mice had positive cultures. Consistent with our earlier reports where FL minimally reduces bacterial burden at the site of infection (2.5X reduction in wound bacterial counts) but prevents the infection from spreading [19], FL treatment did not eliminate the large inoculum from the wound, but did prevent systemic dissemination. pDC depletion prior to FL treatments caused a 2.5X increase in wound bacterial counts, but a 400X increase in bacterial counts in the spleen (Fig. 2). FL appears to increase the ability of the immune system to contain the infection locally in a pDC-dependent manner.

### pDCs are required for FL-mediated enhancement of neutrophil response to infection

**Fig. 3:**
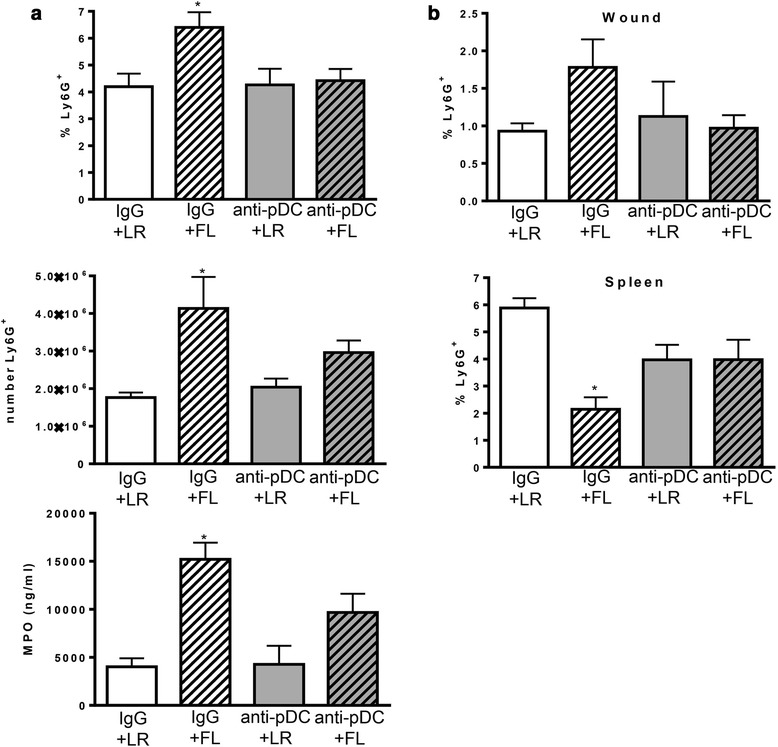
pDCs are required for FL to increase neutrophil responses to infection. Mice were injected with control IgG or anti-pDC antibodies prior to initiation of FL or LR control treatment. Tissues were harvested 3 days following wound inoculation. **a** The percentages (top panel) and numbers (middle panel) of Ly6G^+^ neutrophils and MPO levels (bottom panel) in the wound-draining lymph nodes. *significantly different from both LR groups, *p* ≤0.05, (**b**) Percentages of Ly6G^+^ neutrophils in wounds and spleens following infection are shown. *significantly different from IgG + LR, *p* ≤0.05. *n* = 3–6 mice per group

**Fig. 4:**
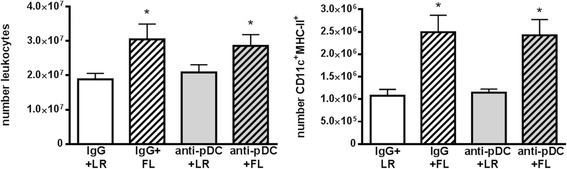
pDC depletion does not alter total leukocyte or cDC numbers. Mice were treated as in Fig. 3. *significantly different from both LR groups, *p* ≤0.05, *n* = 5 mice per group

The primary mechanism by which FL treatments protect against burn-associated sepsis is through enhancement of neutrophil responses to infection, resulting in limited dissemination and accelerated resolution of infection [13]. As previously reported, treatment of mice with FL after burn injury significantly increased the numbers of neutrophils responding to burn wound infection. At 3 days after wound inoculation, the percentages of neutrophils in the wound-draining lymph nodes were higher in mice treated with FL, compared to all other groups (Fig. 3a). Additionally, total numbers of neutrophils were significantly higher in wound-draining lymph nodes of FL-treated mice (~2.3-fold) compared to LR-treated mice, as were levels of myeloperoxidase (MPO) (~3.8-fold). However, pDC depletion prior to FL treatment reduced MPO and the numbers and percentages of neutrophils in the lymph nodes to levels that were no longer significantly different from the control treatment (LR) group. This indicates that pre-existing pDCs must be present during FL treatment for the subsequent enhancement of neutrophil responses to wound infection initiated several days later. Neutrophils in infected wounds were approximately 2-fold higher after FL treatment compared to LR treatment, but not if pDCs had been depleted prior to FL treatment. While the differences between treatment groups were not statistically significant for wound measurements (*p* = .07), the trend was the same as that seen in the wound-draining lymph nodes (Fig. 3b). The opposite trend was observed in the spleen. In response to wound infection, there was a concomitant decrease in spleen neutrophils in FL-treated mice (~3-fold compared to LR), as neutrophil migration to infected wounds increased, and both trends were attenuated if pDCs were not present during treatment. The effects of pDC depletion during FL treatment were specific for neutrophils, as overall cellularity and cDC numbers were not similarly decreased by pDC depletion. As previously reported [19, 20], treatment of mice with FL after burn injury significantly increased overall cellularity and the numbers of cDCs in wound-draining lymph nodes (Fig. 4). Lymph nodes of FL-treated mice had significantly more leukocytes (~1.6-fold) and DCs (~2.5-fold) than lymph nodes of LR-treated mice, an effect that was not altered by pDC depletion.

**Fig. 5:**
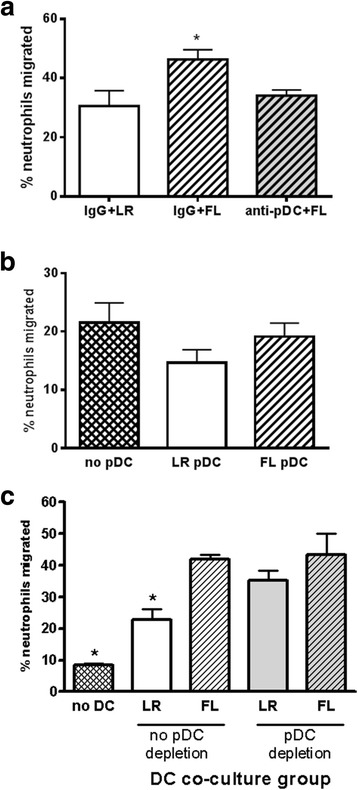
pDCs are required, indirectly, for FL-mediated enhancement of neutrophil chemotaxis. **a** Neutrophils were harvested from burned mice treated with FL in the presence and absence of pDCs (IgG + FL, anti-pDC + FL) and migration towards fMLP was measured. *significantly different from other groups, *p* <0.05, *n* = 6–15. **b** PDCA-1^+^ pDCs were isolated from burned mice that had been treated in vivo with control LR or FL and were co-cultured overnight with neutrophils that had been harvested from burned, non-treated mice. Migration to fMLP was subsequently assessed. *n* = 5–15. **c** CD11c^+^ DCs were isolated from burned mice that had been depleted of pDCs or not at the start of FL treatment as in Fig. 1. Neutrophils were harvested from burned mice and co-cultured with DCs overnight prior to measurement of migration towards fMLP. *significantly different from all other groups, *p* <0.05, *n* = 4–6

Enhanced neutrophil responses to infection in FL-treated mice is associated with an increased migratory capacity of neutrophils, demonstrated by greater in vitro chemotaxis of neutrophils freshly harvested from FL-treated mice compared to control-treated mice [13]. As shown in Fig. 5a, chemotaxis to fMLP was significantly greater in neutrophils that had been freshly harvested from mice treated with FL (IgG + FL) compared to neutrophils from LR (IgG + LR)-treated mice. However, if pDCs had been eliminated prior to initiation of FL treatment in vivo, subsequent in vitro chemotaxis of isolated neutrophils was no longer increased by FL.

In vitro, DCs can directly enhance neutrophil migratory capacity. Specifically, the ability of neutrophils to migrate to a common stimulus in vitro is significantly increased after exposure to DCs in overnight culture. FL further enhances this effect of DCs on neutrophil migratory capacity [13]. To determine if the pDC subset may directly enhance neutrophil migration, we similarly co-cultured pDCs with neutrophils prior to assessment of neutrophil migration. Co-culture of neutrophils with pDCs did not enhance neutrophil migration (Fig. 5b). Migration of neutrophils exposed to pDCs from FL-treated mice was not different when compared to that of neutrophils that had not been previously cultured with pDCs. In fact, exposure of neutrophils to pDCs from LR-treated mice appeared to slightly decrease neutrophil migration capacity, but the difference between groups was not statistically significant.

The finding that pDCs and cDCs are both necessary for FL enhancement of neutrophil response to infection in vivo [13], yet pDCs do not directly enhance neutrophil migration, indicates that FL induces effects in pDCs that indirectly modulate neutrophils. It has been reported that pDCs can act as “helper” cells to cDCs to enhance cDC-initiated immune responses [17, 18]. We therefore investigated the possibility that pDCs may “help” cDCs to enhance neutrophil migration capacity. As demonstrated in Fig. 5c, and as previously reported, co-culture of neutrophils with CD11c^+^ DCs from control (LR)-treated mice significantly increased subsequent neutrophil chemotaxis (2.7-fold) when compared to neutrophils that had not been previously cultured with DCs. Prior incubation of neutrophils with CD11c^+^ DCs from FL-treated mice further increased their migration 1.8-fold when compared to neutrophils exposed to LR cDCs, and 5-fold when compared to migration of neutrophils that had not been previously cultured with DCs. Depletion of pDCs at the initiation of in vivo FL treatment did not affect the ability of the remaining cDCs to enhance neutrophil chemotaxis. Depletion of pDCs in LR-treated mice actually increased the ability of cDCs to promote neutrophil migration, but had no effect on FL enhancement of migration. These data indicate that pDCs do not serve as helpers of cDCs in their function of modulating neutrophil migration capacity.

## Discussion

In earlier studies, we found that FL has the potential to be used as a prophylactic treatment after burn injury to increase resistance to subsequent infections. When administered before or after burn injury, FL increases DC numbers, restores Th1 cytokine responses, prevents bacterial growth, dissemination of infection and systemic inflammation, and increases survival following infection [8, 20]. A recent study shows that FL treatment also enhances adaptive immune responses through attenuation of T cell dysfunction after burn wound sepsis [21]. FL-altered DCs have been shown to directly induce several of these beneficial alterations. Adoptive transfer of CD11c^+^ DCs from FL-treated mice significantly increases survival after wound infection in non-treated recipient mice whereas equivalent numbers of DCs from control-treated mice do not improve survival. Conversely, depletion of CD11c^+^ DCs prevents FL-mediated protection against infection [13]. However, FL stimulates the production of both pDCs and cDCs, distinct populations of cells that contribute to immune responses by distinct mechanisms. As pDCs in mice express low levels of CD11c, it is possible that pDCs, although comparatively lower in numbers than cDCs, could have contributed to the previously reported effects of FL. Therefore, anti-mouse pDCA-1 was used here to specifically deplete pDCs, but not cDCs, during FL treatment. We found that pDCs are required for the beneficial effects of FL in vivo, but within a different time frame than the requirement for cDCs. Specifically, we previously observed that either depletion of pre-existing CD11c^+^ DCs at the start of FL treatment or depletion of FL-expanded CD11c^+^ DCs after FL treatment, at the time of infection, abrogates the protective effects of FL in burn-injured mice [13]. However, we show here that the pDC subset is not required at the time of infection, but is required at the time of treatment for FL to enhance neutrophil responses to infection, bacterial clearance, and survival after wound infection (Figs. 1, 2 and 3). This indicates that FL has early effects on pre-existing (prior to FL-induced expansion) pDCs that impact subsequent responses to infection, perhaps through the priming or activation of other immune cells.

A primary mechanism by which FL enhances resistance to infection after burn injury is through enhancement of neutrophil migration to sites of infection [13]. This was confirmed here, as treatment of mice with FL increases the numbers of neutrophils responding to burn wound infection, indicated by the increased presence of neutrophils and higher levels of MPO in the wound-draining lymph nodes of infected mice treated with FL (Fig. 3). Preexisting pDCs are required during FL treatment for these in vivo effects, as their depletion abolishes the effects of FL on neutrophil responses. At the same time, splenic neutrophils significantly decrease during infection in FL-treated mice, and this is also dependent on preexisting pDCs. This suggests that splenic neutrophils are somehow primed by FL in a pDC-dependent manner for a rapid response to infection. Indeed, freshly isolated neutrophils from FL-treated mice show increased chemotaxis in vitro when compared to neutrophils isolated from LR-treated mice, but only if pDCs were present during in vivo FL treatment (Fig. 5a). While the mechanism by which preexisting FL-stimulated pDCs prime neutrophils for rapid migration is not known, our data indicate that this is mediated indirectly. Co-culturing neutrophils with pDCs does not increase subsequent migration (Fig. 5b), whereas the direct exposure of neutrophils to cDCs does increase neutrophil migratory capacity, an effect that is enhanced by FL (Fig. 5c). The finding that pDC depletion abrogates the effects of FL on neutrophil responses in vivo (Fig. 3) indicates that cDCs are not able to effectively enhance neutrophil migratory capacity if pDCs are not present during treatment.

pDCs have been shown by others to enhance some cDC functions. Specifically, cDC interactions with pDCs via CD40:CD40 ligand on cDCs and pDCs, respectively, are requisite for CpG DNA-stimulated IL-12 production by cDCs [18]. As both pDCs and cDCs are required for FL enhancement of neutrophil migration to infection in vivo [13], and cDCs but not pDCs directly enhance neutrophil migratory capacity, we reasoned that pDCs may prime or signal cDCs in their capacity as enhancers of neutrophil migration. However, depletion of pDCs at the start of FL treatment, which abrogated enhanced neutrophil responses in vivo, did not affect the subsequent ability of cDCs to directly enhance neutrophil chemotaxis in vitro (Fig. 5c). Therefore, the mechanisms remain to be identified. It should be noted that the consequence of pDC depletion was specific and not a global reduction in cellular responses to infection as cDCs and overall cellularity in the wound draining lymph nodes were not similarly reduced by prior pDC depletion (Fig. 4). Also, anti-PDCA-1 antibodies do not directly deplete neutrophils themselves and do not cause a decrease in neutrophil numbers in the absence of infection. Additionally, the effects of pDCs on neutrophil responses are specific to FL treatment, as depletion of pDCs in LR-treated mice does not decrease neutrophil responses to infection in vivo (Fig. 3). This indicates that activation of pDCs by FL induces unique responses that influence neutrophil migration in vivo.

This study is limited by the lack of an identified underlying mechanism. The unusual time frame during which pDC presence in vivo is required for FL to protect against later infections suggests that the underlying mechanisms are likely complex and challenging to dissect. Specifically, pDCs were depleted on day 1 and FL treatments initiated on day 2 (Fig. 1), and wounds were not inoculated until day 5. The effect on neutrophil responses to infection, specifically, decreased neutrophil presence in wound-draining lymph nodes, was demonstrated 7 days after pDC depletion (Fig. 3). A possible explanation of these findings is that FL directly stimulates pre-existing pDCs early during treatment to produce a chemotactic factor that mobilizes neutrophils and/or cDCs and facilitates neutrophil-cDC interactions. In this scenario, the depletion of cDCs would be sufficient to abolish the protective effects of FL because neutrophil migratory capacity would not be enhanced via cDC-neutrophil interactions [13]. Likewise, the depletion of pDCs in vivo would also be sufficient to prevent the effects of FL, as the signal that facilitates cDC-neutrophil interactions in vivo would not be induced (as in Fig. 3). However, co-culture of neutrophils with cDCs would still enhance neutrophil migratory capacity as this is not dependent on pDCs (as in Fig. 5c). While the data presented here are consistent with that scenario, further studies are needed to dissect this complex in vivo response to infection and FL treatment. We have investigated the potential contribution of individual cytokines that are known to play a role in neutrophil mobilization and maturation/activation. While we found that FL has effects on some of these mediators, it also modulates the production of numerous other cytokines, especially those associated with Th1 responses during infection [20]. To date, we have been unable to identify a specific mediator that is induced by FL only in the presence of preexisting pDCs. Similarly, we have been unable to identify neutrophil phenotypes that are induced by FL only in the presence of pDCs. The early responses to burn injury are complex and dynamic and involve the mobilization of numerous innate immune cells, including neutrophils and DCs, from multiple tissues to sites of injury [22]. Additionally, there is a systemic inflammatory response to burn injury, where numerous mediators are released into the circulation [23]. Therefore, it is possible that multiple factors induced by FL and burn injury may act synergistically to induce these beneficial, pDC-dependent effects on neutrophil responses to infection. Future studies will investigate multiple factors that may act in concert to mediate these effects of FL.

## Conclusions

Although the underlying mechanisms by which pDCs indirectly mediate the protective effects of FL have yet to be identified, this study provides some novel findings. The ability of FL to enhance neutrophil responses to burn wound infection, bacterial clearance, and survival is dependent upon both pDCs and cDCs. While pDCs are required for FL to enhance neutrophil responses in vivo, pDCs do not directly enhance the capacity of neutrophils to migrate, and do not serve as “helper” cells to cDCs in this capacity. Regardless of the mechanisms, the significant protection against burn wound infection afforded by FL supports the potential utility of FL as a treatment to decrease infections in burn patients.

## Methods

### Mice

All animal procedures were approved by the Institutional Animal Care and Use Committee at the University of Texas Medical Branch and meet the National Institutes of Health guidelines for the care and use of experimental animals. Male, BALB/c mice, 6–10 weeks of age, were given a preemptive dose of buprenorphine (0.1 mg/kg, s.c.), then deeply anesthetized with isofluorane 30 min later. Dorsal and lateral surfaces were shaved with clippers, and mice were placed on their backs in a protective template that exposed approximately 35% of the total body surface area TBSA. A non-lethal full-thickness injury was induced by immersion of the exposed skin in 97–99 °C water for 10 s, and fluid resuscitation (lactated Ringers solution, LR, 2 cc, i.p.) was administered. Mice were monitored twice daily for signs of discomfort and administered buprenorphine up to every 12 h if needed. Recombinant FL was provided by Celldex Therapeutics (Needham, MA), and was injected once daily (10 μg in 0.1 cc LR, i.p.). Control treatment mice received the same volume of LR according to the same protocol. FL treatment was initiated on either the day of or after burn injury and continued for 4 days. We have demonstrated that FL treatments can be started immediately after injury or several days later with similar efficacy [19]. Anti-plasmacytoid DC antigen-1 (pDCA-1, or anti-pDC, Miltenyi Biotec) or control IgG was injected for two consecutive days (500 μg, i.p.), beginning on the day before FL treatment (early depletion) or on the last day of FL treatment (late depletion). At the end of experiments, mice were humanely euthanized according to the AVMA Guidelines of Euthanasia, via cervical dislocation under isoflurane (5% in air) anesthesia.

### Infection

*P. aeruginosa* (ATCC #19660, Manassas, VA), a common source of wound infections in burn patients, was grown in tryptic soy broth, diluted in sterile saline, and applied topically to the surface of the burn wound. Wounds were inoculated 4 or 5 days after burn injury with 10^4^ or 5x10^4^ CFU, respectively. Survival was monitored for 14 days post-inoculation. For determination of cfu/g tissue wet weight, spleen and skin samples were weighed, homogenized in sterile saline and serial dilutions plated and incubated overnight on tryptic soy agar.

### In vitro chemotaxis

Minced spleens and wound-draining lymph nodes (axillary, inguinal) were mechanically dissociated and passed through a 70 μm strainer. Red blood cells were lysed (RBC Lysing Buffer Hybri-Max, Sigma-Aldrich), cell suspensions were washed, and neutrophils were isolated using Ly6G (1A8) microbeads and Miltenyi magnetic separation columns according to manufacturer’s directions (Miltenyi Biotech). Neutrophils were plated in the upper chambers of polycarbonate transwell filters (5 μm pores, Thermo Fisher Scientific) with 10^−6^ M fMLP (Sigma) in the lower chamber, and incubated at 37 °C for 2–3 h. Cells in the lower chamber were detached by addition of 7 mM EDTA (Sigma) and counted using a hemocytometer and trypan blue. In co-culture experiments, CD11c^+^ DCs or PDCA-1^+^ pDCs were co-cultured with neutrophils overnight in RPMI-1640 supplemented with 10% fetal bovine serum and antibiotics at a ratio of (1:10 DC to neutrophils) prior to the chemotaxis assay.

### Flow cytometry

Spleen and lymph node single cells suspensions were prepared as described above. Wound cells were liberated by incubation in 1 mg/ml collagenase D for 30 min at 37 °C in a shaking incubator. Single cell suspensions were incubated with 0.5 mg/ml Fc block and 0.5 μg fluorescence-conjugated antibodies against specific surface proteins for 20 min at 4 °C, then washed in PBS. Cells were fixed and permeabilized using Cytofix/Cytoperm (BD Biosciences) according to manufacturer’s instructions, washed in BD Perm/Wash, then incubated with 0.5 μg fluorescence-conjugated antibodies. After washing, cells were analyzed on a Guava® easyCyte 8HT flow cytometer, using InCyte software (Millipore). Antibodies against B220, CD11c, and Ly6G were from BD.

### Myeloperoxidase (MPO)

Wound-draining lymph nodes were homogenized in lysis buffer (200 mM NaCl, 5 mM EDTA, 10 mM Tris, 10% glycerol, 1 mM PMSF). MPO was measured by ELISA per manufacturer’s instructions (Cell Sciences).

### Statistics

Analyses were performed using GraphPad Prism 6.00 for Windows, GraphPad Software, La Jolla California USA, www.graphpad.com. Survival curves were estimated by Kaplan-Meier method and compared by log-rank test. Unpaired, two-tailed Student’s t-tests compared two groups. Multiple groups were analyzed by ANOVA/Tukey-Kramer multiple comparisons test. *p* ≤ 0.05 was considered significant.

## Abbreviations

cDC: Conventional dendritic cell
DC: Dendritic cell
FL, Flt3 ligand: fms-like tyrosine kinase-3 ligand
Flt3R: fms-like tyrosine kinase-3 ligand receptor
MPO: Myeloperoxidase
pDC: Plasmacytoid dendritic cell
